# Cancer cell line microarray as a novel screening method for identification of radioresistance biomarkers in head and neck squamous cell carcinoma

**DOI:** 10.1186/s12885-021-08618-6

**Published:** 2021-07-29

**Authors:** Johannes Routila, Karri Suvila, Reidar Grénman, Ilmo Leivo, Jukka Westermarck, Sami Ventelä

**Affiliations:** 1 Turku Bioscience Centre, University of Turku and Åbo Akademi university, Turku, Finland; 2 Department for Otorhinolaryngology – Head and Neck Surgery, Turku University Hospital and University of Turku, Kiinamyllynkatu 4-8, 20521 Turku, Finland; 3 Department for Otorhinolaryngology, Satakunta Central Hospital, Pori, Finland; 4 Institute of Biomedicine, University of Turku, Kiinamyllynkatu 10, 20520 Turku, Finland

**Keywords:** Radiosensitivity, Radioresistance, Head and neck cancer, Oct4, Stemness

## Abstract

**Background:**

Currently, no clinically useful biomarkers for radioresistance are available in head and neck squamous cell carcinoma (HNSCC). This study assesses the usefulness of Cell Line Microarray (CMA) method to enhance immunohistochemical screening of potential immunohistochemical biomarkers for radioresistance in HNSCC cell lines.

**Methods:**

Twenty-nine HNSCC cell lines were cultured, cell pellets formalin-fixed, paraffin-embedded, and arrayed. Radioresistance features of the cell lines were combined to immunohistochemical stains for p53, NDFIP1, EGFR, stem cell marker Oct4, and PP2A inhibitor CIP2A.

**Results:**

Expression of p53, EGFR or CIP2A did not indicate intrinsic radioresistance in vitro. Stem cell marker Oct4 nuclear positivity and NDFIP1 nuclear positivity was correlated with increased intrinsic radioresistance.

**Conclusion:**

The usefulness of CMA in analysis of HNSCC cell lines and discovery of biomarkers is demonstrated. CMA is very well adapted to both testing of antibodies in a large panel of cell lines as well as correlating staining results with other cell line characteristics. In addition, CMA-based antibody screening proved an efficient and relatively simple method to identify potential radioresistance biomarkers in HNSCC.

**Supplementary Information:**

The online version contains supplementary material available at 10.1186/s12885-021-08618-6.

## Background

Radiotherapy has a major role in the treatment of head and neck squamous cell carcinoma (HNSCC). Albeit the general radiosensitivity of HNSCC, radioresistance of a subset of the tumors is a major clinical problem requiring further study both for an enhanced mechanistic understanding of radioresistance, and to overcome clinical radioresistance in patient therapy. Putative mechanisms responsible for clinical radioresistance include hypoxic environment, EGFR-pathway alterations, epithelial-mesenchymal transition, deregulation of p53, angiogenesis, and cancer stem cells [1]. Despite extensive characterization, investigations into clinical biomarkers for HNSCC radioresistance have proven disappointing [2, 3].

The inability to correctly identify clinically significant molecular events in cell line studies remains a significant problem, since virtually all in vivo cancer studies are preceded by in vitro cell line investigation into cancer behavior and characteristics. The genomic and molecular diversity of different cell lines complicates the selection of cell lines for in vitro studies. Behavior of different cell lines may vary according to the planned experiment e.g. due to differences in protein expression or genomic features. Thus, selection from the multitude of available cell lines happens often by trial and error. Thus, there is a high demand for high-throughput screening method for cell line characteristics.

Tissue microarray (TMA) methods have proven to be an excellent tool for profiling and screening tumor samples [4, 5]. Recently, to answer the demand for high-throughput screening methods for cell line characteristics, cell-based microarrays have been introduced [6, 7]. The cell microarray (CMA) offers a powerful tool in the evaluation of cell lines. The absence of interference of extracellular matrix facilitates the study of novel antibodies or genomic methods and allows for more reliable specificity analysis of antibodies. The validity of stainings can be assessed by comparing the results to previously acquired data. The included cell lines can be cultured in various conditions, e.g., to include shRNA-treated cells to aid in validation of analysis of proteins of interest. The function of CMA as a fixed, paraffin-embedded biobank of cell lines is of special importance, as it can be stored virtually without limits for later use after the relatively tedious original cell cultures.

In this study, we aimed to assess a carefully selected panel of five immunohistochemical stains of putative radiotherapy biomarkers – p53, EGFR, Oct4, NDFIP1 and CIP2A – in head and neck cancer cell lines. Mutations of the tumor suppressor p53 occur frequently in HNSCC tumors and cell lines [8, 9]. While the exact type of p53 genetic alteration may predict HNSCC radioresistance, the role of p53 expression is unclear [10]. The role of epidermal growth factor receptor (EGFR) for therapy resistance of HNSCC has been an active field of investigation, since EGFR inhibitor cetuximab was introduced and approved for use in HNSCC [11, 12]. The results of cetuximab-based chemo- or chemoradiotherapy have proven disappointing, despite that EGFR expression seems to associate with HNSCC clinical radioresistance [12]. Oct4 is a stem cell marker which has been linked to radioresistance through cancer stem cell phenomenon and epithelial-mesenchymal transition in several previous studies [13–15]. The prognostic relevance of CIP2A to HNSCC has previously been established [13, 16]. CIP2A mediates radioresistance in HNSCC and colorectal cancer, and is linked to several oncogenic signaling mechanisms such as c-Myc, p53, EGFR, mTOR signaling and Oct4 [17, 18]. Nedd4 family interacting protein NDFIP1 is previously linked to radioresistance of HNSCC through PTEN regulation [19]. Overexpression of NDFIP1 RNA is associated with an unfavorable prognosis in The Cancer Genome Atlas (TCGA) RNA-Seq data [20].

## Methods

### Cell lines

The included 26 UT-SCC cell lines are summarized in Table 1. UT-SCC cell lines were established as previously reported from various HNSCC tumors according to ethical approval by the Ethics Committee of the Hospital District of Southwest Finland as well as patient informed consent [21]. All experiments were carried out according to institutional guidelines and the Declaration of Helsinki. The clinical course of every donor patient’s disease was monitored and clinical data from patient was gathered [22, 23]. Cell lines were cultured at 37 °C and 5% CO_2_ in Dulbecco’s Modified Eagle’s Medium (DMEM), with 10% FCS, glutamine and antibiotics (penicillin and streptomycin). The intrinsic radioresistance of UT-SCC cell lines was previously determined using the 96-well clonogenic assay for radioresistance [8, 9, 22]. In short, the cells were cultured, plated on 96-well plates, exposed to photon irradiation doses ranging from 0.75 to 7.5 Gy the number of dividing cells calculated, and area under the curve (AUC) of cell survival was calculated. For analysis, the mean inactivation dose defined as the AUC of survival curve is used.

**Table 1:** The main characteristics of the UT-SCC cell lines included in the study

Cell line	Sex	Age	Primary tumor site	Grade	T	N	M	Specimen site	Type	Radioresistance, AUC
UT-SCC-2	m	60	floor of mouth	2	4	1	0	floor of mouth	primary	1.8 ± 0.2
UT-SCC-5	m	58	tongue	2	1	1	0	tongue	primary	2.3 ± 0.3
UT-SCC-7	m	67	temporal skin	2	1	0	0	neck	metastasis	2.0 ± 0.2
UT-SCC-8	m	42	supraglottic	1	2	0	0	larynx	primary	1.9 ± 0.1
UT-SCC-9	m	81	glottic	1	2	1	0	neck	metastasis	1.4 ± 0.1
UT-SCC-14	m	25	tongue	2	3	1	0	tongue	primary	1.7 ± 0.3
UT-SCC-16A	f	77	tongue	3	3	0	0	tongue	primary	1.8 ± 0.1
UT-SCC-17	m	65	supraglottic	3	2	0	0	sternum	metastasis	1.8. ± 0.1
UT-SCC-19A	m	44	glottic	2	4	0	0	larynx	primary	1.7 ± 0.1
UT-SCC-20A	f	58	floor of mouth	2	1	0	0	floor of mouth	primary	2.1 ± 0.2
UT-SCC-24A	m	41	tongue	2	2	0	0	tongue	primary	2.6 ± 0.3
UT-SCC-25	m	50	tongue	1	2	0	0	tongue	recidive	
UT-SCC-30	f	77	tongue	1	3	1	0	tongue	primary	2.0 ± 0.1
UT-SCC-32	m	66	tongue	2	3	0	0	tongue	primary	1.7 ± 0.3
UT-SCC-34	m	63	supraglottic	1	4	0	0	larynx	primary	2.0 ± 0.1
UT-SCC-36	m	46	floor of mouth	3	4	1	0	floor of mouth	primary	2.2 ± 0.2
UT-SCC-45	m	76	floor of mouth	3	3	1	0	floor of mouth	primary	2.0 ± 0.1
UT-SCC-46A	m	62	retromolar gingiva	3	1	0	0	gingiva	primary	1.6 ± 0.1
UT-SCC-47	m	78	floor of mouth	3	2	0	0	floor of mouth	primary	2.0 ± 0.2
UT-SCC-50	m	70	glottic	3	2	0	0	larynx (rT2N0)	recidive	
UT-SCC-60B	m	59	tonsil	1	4	1	0	neck	metastasis	2.2 ± 0.3
UT-SCC-72	m	50	mandibular gingiva	2	4	2	0	gingiva	primary	2.8 ± 0.2
UT-SCC-74A	m	31	tongue	2	3	1	0	tongue	primary	
UT-SCC-76A	m	52	tongue	2	3	0	0	tongue	primary	2.5 ± 0.2
UT-SCC-79A	f	80	parotid	2	0	2	0	parotid	metastasis	2.4 ± 0.2
UT-SCC-79B	f	80	parotid	2	0	2	0	neck	metastasis	2.5 ± 0.1

### shRNA cell lines

Three previously established stable shRNA-transfected UT-SCC cell lines were included, two shCIP2A-silenced variants of UT-SCC-24A and one shCIP2A-silenced variant of UT-SCC-14A. shRNA cell lines were generated using pGIPZ lentiviral vectors consisting of a GFP tag and puromycin resistance (Open Biosystems). Cells transfected with lentiviral vector pGIPZ.NS shRNA containing non-silencing shRNA served as control cells expressing high CIP2A. Two stable cell lines for low CIP2A expression were generated using pGIPZ.shRNA (#556) and pGIPZ.shRNA (#557) containing targeting CIP2A antisense sequences TACATCAGCAGCAAGTTTG and TACTCAATGTCTTTATGTG, respectively. Lentivirus production followed the standard protocol. At the time of transduction, cell line confluency was approximately 40–50%. After infection, the cells were selected for puromycin resistance, and if the number of GFP-positive cells was low, the cells were sorted with FACS for further experiments. Finally, all cell lines were tested negative for replication competent viruses (RCV test) as well as for Mycoplasma, Acholeplasma, Entomoplasma and Spiroplasma (The MycoAlert™ Mycoplasma Detection kit, Lonza). Western blot using anti-CIP2A antibody (dilution 1:1000, 2G10-3B5, sc-80,659, SantaCruz) was used to confirm the successful silencing of CIP2A expression (Fig. 4A).

### CMA construction

**Fig. 1:**
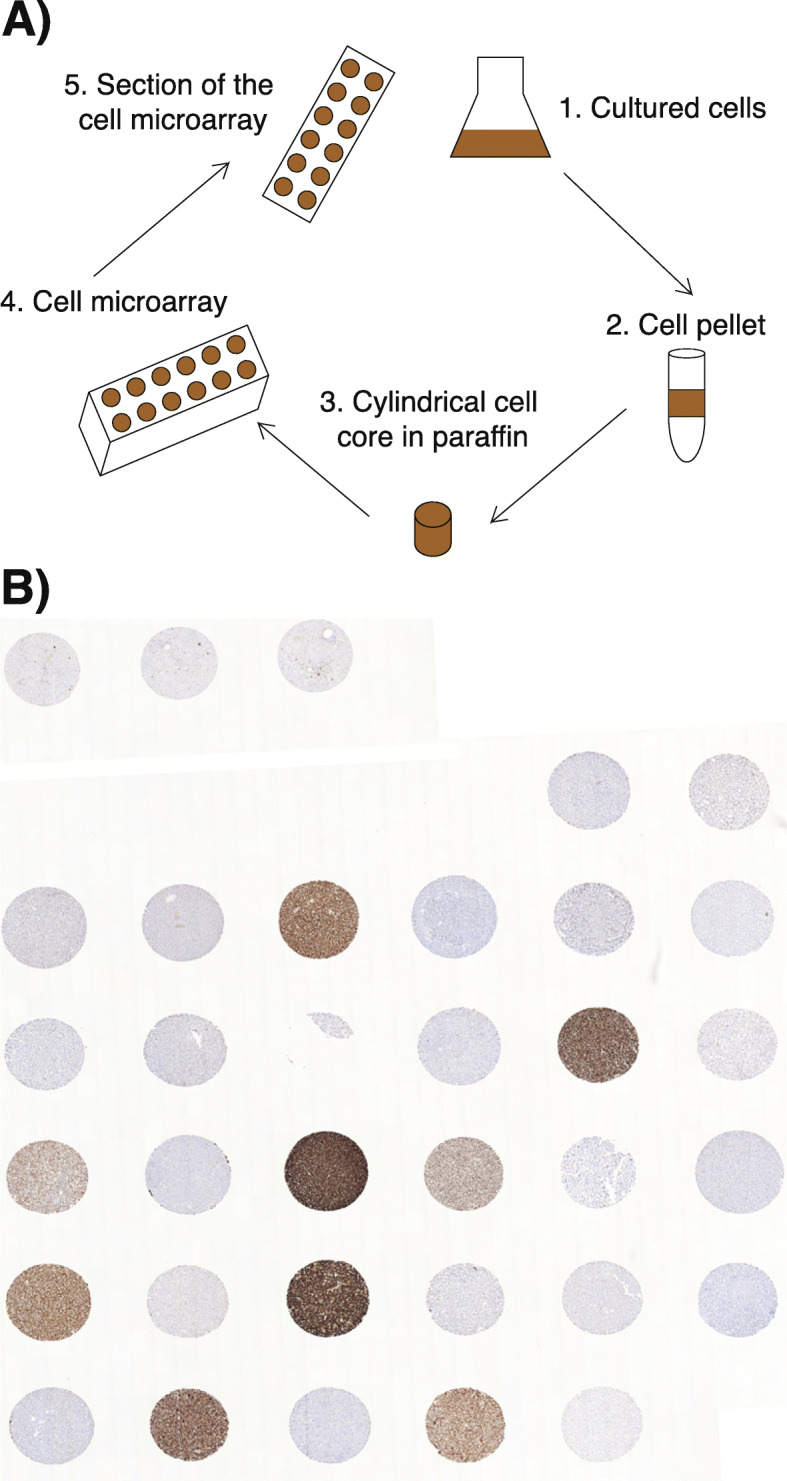
**A** Schematic representation of the CMA construction. Cores from each cell line block are combined into a single paraffin-embedded array. **B** Overview of the completed CMA (p16 immunohistochemical staining)

Cultured cells were harvested by trypsinization before pelleting the cells. Approximately 40 × 10^6^ cells were used for each cell pellet. Pellets were washed with PBS and resuspended in 120–160 μl of 10% neutral-buffered formalin. Cells were then added into a microfuge tube containing a conical fill made of 2% agarose in PBS. After the spin (1000 rpm × 5 min) the supernatant was removed and 10 ml of buffered formalin was added for 48 h, after which the pellets were stored in PBS (+ 4 °C). Microfuge tubes were cut open, the pellet transferred into a tissue cassette, and submitted for paraffin embedding (Fig. 1). A microarray was assembled by using the services of Auria Biobank (Auria Biobank, Turku, Finland). The formalin-fixed, paraffin-embedded cell pellets were cut for 6 μm sections and haematoxylin-eosin stained. The slides were scanned and annotated for 0.6 mm cores using Pannoramic Viewer software. The annotated cores were combined into duplicate receiver blocks using TMA Grand Master (3D Histech). Samples from normal human liver were included for orientation.

### Immunohistochemistry

Six micrometer sections were obtained from the final CMA and TMA block for IHC stainings. p53 and EGFR immunohistochemical stainings was obtained from local clinical pathology department laboratory and carried out in Ventana staining automate. CIP2A immunohistochemical staining was carried out after protocol optimization in Ventana BenchMark XT staining automate (Ventana, Tucson, AZ) with OptiView DAB kit and with 64-min CC1 preparation and 32-min antibody incubation. Mouse monoclonal anti-CIP2A antibody (dilution 1:25, 2G10-3B5, sc-80,659, SantaCruz) was used. For PME-1, SET, LIMA1, NDFIP1, and Oct4 stainings, immunohistochemical stainings were done as previously described [13, 24]. The antibodies used were rabbit polyclonal anti-SET (H-120) antibody sc-25,564 (diluted 1:1000, Santa Cruz Biotechnology), mouse monoclonal anti-PME-1 (B-12) antibody sc-25,278 (diluted 1:1000, Santa Cruz Biotechnology), rabbit polyclonal anti-NDFIP1 antibody HPA009682 (diluted 1:500, Sigma-Aldrich), and mouse monoclonal anti-Oct4 antibody sc5279 (diluted 1:200, Santa Cruz Biotechnology).

### Scoring of immunohistochemistry

All stainings were analyzed independently by at least two authors (JR, KS, IL, SV), and contradictory cases were discussed until consensus was reached. For microscopic photographs, slides were scanned using Pannoramic 250 Flash slide scanner. Using the CaseViewer software, photographs of either 10-fold or 20-fold magnification were exported in 300 dpi quality. Images were cropped in image editing software, while no color adjustments were performed.

p53 staining was analyzed using a previously established 3-tier system consisting of deletion-like absence of p53 staining, wild-type pattern of staining, and mutation/amplification-associated aberrant-type overexpression. EGFR staining was scored in a 4-tier system (0, +, ++, +++) for cytoplasic/membraneous staining pattern. For CIP2A, the intensity of the cytoplasmic/membraneous staining was scored on a scale of 1 to 3 as weak/negative, intermediate or strong, taking into account the number of positive cells. In patient samples, CIP2A was uniformly present and was scored similarly based on cytoplasmic/membraneous staining intensity. Oct4 immunostaining was analyzed for the presence of positive nuclei. Oct4 positivity was present only in a subpopulation of cells, and thus the presence of strong nuclear immunoreactivity of individual cells was regarded positive. NDFIP1 staining was scored positive, when strong, uniform nuclear staining pattern was present, whereas cytoplasmic staining was not taken into account. A three-tier scoring system was used for uniform nuclear PME-1 and SET staining [24].

### Statistical analysis

Cell line data, patient data, and results of immunohistochemistry were entered in SPSS 25 software. Dependence of cell line radioresistance to the staining results was analyzed using General Linear Model statistics. Both main effects and interactions were observed. Estimated marginal means were calculated and 95% confidence intervals determined using bootstrapping. Correlation was determined by Spearman’s method. Throughout, *p* value < 0.05 was deemed significant.

## Results

### CMA construction

For the CMA construction, UT-SCC cell lines with previous data on radiosensitivity were preferentially selected. Altogether 26 UT-SCC cell lines were included, the majority of which were derived from male patients with oral cavity cancer (Table 1). Six cell lines were derived from metastatic samples, and two cell lines from recurring cancers. In addition, three stable CIP2A shRNA-silenced cell lines were included. The cell lines were cultured until a sufficient number of cells was obtained, whereafter cells were pelleted, fixed in formalin and embedded in paraffin. The following formalin-fixed, paraffin-embedded cell blocks were annotated, and a CMA was arrayed. The CMA construction is demonstrated in Fig. 1A and an example of immunohistochemical assessment in Fig. 1B.

### Association of biomarker staining intensities and intrinsic radioresistance

**Fig. 2:**
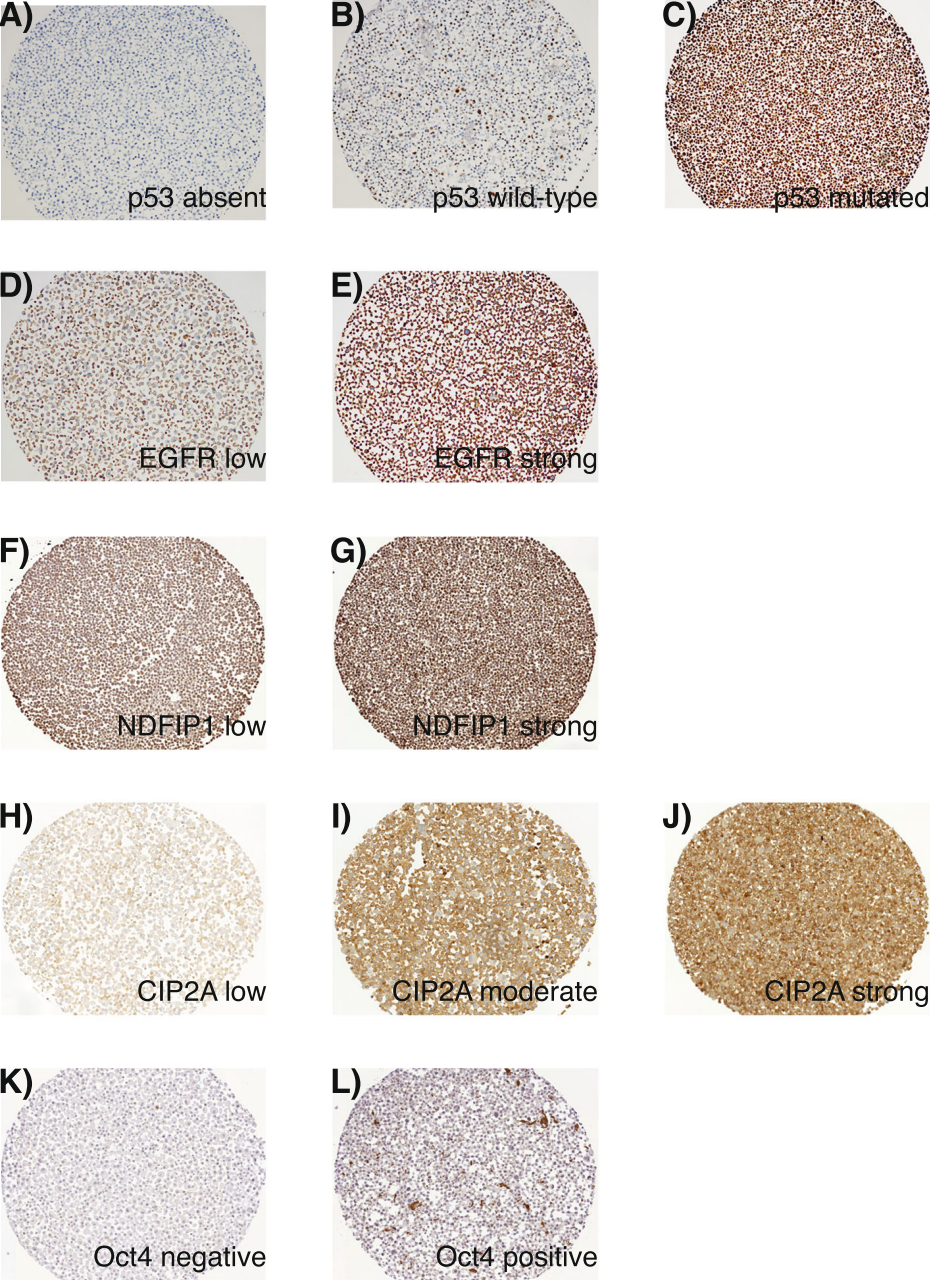
Representative images of immunohistochemical stains of UT-SCC cell line CMA. p53 **A** absent (deletion-type staining), **B** wild-type, **C** mutated (mutation/amplification type staining). EGFR (**D**) low, and (**E**) strong cytoplasmic/membraneous staining. NDFIP1 (**F**) low nuclear staining, and (**G**) strong positive nuclear stain, despite only negligible difference in cytoplasmic staining. CIP2A (**H**) low, (**I**) moderate, and (**J**) strong cytoplasmic/membraneous staining. Nuclear Oct4 (**K**) negative and (**L**) positive

Immunohistochemical stains for p53, EGFR, Oct4, CIP2A, and NDFIP1 were analyzed (Fig. 2A-L, Table 2). There were significant correlations between Oct4 and NDFIP1 stains (ρ 0.46, *p* = 0.020), but no correlation between the expression of other biomarkers. Patient age and gender or tumor site, T class or nodal positivity were not directly linked with the expression of any investigated biomarkers.

**Table 2:** Immunohistochemistry results of the CMA

Cell line	Primary site	AUC	p53 wt DNA	p53 mutation	Immunohistochemistry					
					p53	LIMA1	EGFR	NDFIP1	Oct4	CIP2A
UT-SCC-2	floor of mouth	1.8 ± 0.2	no	c.275 CCC > CAT	mutated	1	3	0	1	2
UT-SCC-5	tongue	2.3 ± 0.3	no	c.151 CCC > CAT	mutated	2	3	1	1	1
UT-SCC-7	temporal skin	2.0 ± 0.2	yes	c.266 GGA > GAA	mutated	1	3	1	1	3
UT-SCC-8	supraglottic	1.9 ± 0.1	no	c.255 ATC > TTC	mutated	1	3	1	1	2
UT-SCC-9	glottic	1.4 ± 0.1	no	total deletion	absent	1	2	0	0	2
UT-SCC-14	tongue	1.7 ± 0.3	yes	deletion, frameshift	mutated	1	3	0	1	2
UT-SCC-16A	tongue	1.8 ± 0.1		c.110 CGT > TGT / c.232 ATC > AAC	mutated	1	2	0	0	1
UT-SCC-17	supraglottic	1.8. ± 0.1		c.110 CGT > CTT / c.257 CTG > CAG	mutated	2	3	1	1	3
UT-SCC-19A	glottic	1.7 ± 0.1	no	c.285 GAG>AAG	mutated	2	3	0	0	3
UT-SCC-20A	floor of mouth	2.1 ± 0.2	yes	c.248 CGG > TGG	mutated	2	2	1	1	2
UT-SCC-24A	tongue	2.6 ± 0.3	yes	47 bp insertion, frameshift	absent	2	3	1	1	3
UT-SCC-25	tongue		no	c.248 CGG > TGG	mutated	2	3	1	1	2
UT-SCC-30	tongue	2.0 ± 0.1	no	c.282 CGG > CCG	mutated	1	2	0	1	1
UT-SCC-32	tongue	1.7 ± 0.3	no	c.266 GGA > GAA	mutated	1	3	1	0	1
UT-SCC-34	supraglottic	2.0 ± 0.1	yes	total deletion	absent	1	3	1	1	1
UT-SCC-36	floor of mouth	2.2 ± 0.2	no	c.244 GGC > AGC	mutated	1	2	1	1	3
UT-SCC-45	floor of mouth	2.0 ± 0.1			wt	1	3	0	1	1
UT-SCC-46A	retromolar gingiva	1.6 ± 0.1			wt	1	1	0	0	1
UT-SCC-47	floor of mouth	2.0 ± 0.2			absent	2	2	1	1	3
UT-SCC-50	glottic				wt	2	3	1	1	2
UT-SCC-60B	tonsil	2.2 ± 0.3			absent	2	2	0	1	2
UT-SCC-72	mandibular gingiva	2.8 ± 0.2			absent	2	3	1	1	2
UT-SCC-74A	tongue				wt	1	3	0	1	2
UT-SCC-76A	tongue	2.5 ± 0.2			mutated	1	3	0	0	1
UT-SCC-79A	parotid	2.4 ± 0.2	yes	deletion, frameshift	wt	2	3	1	1	1
UT-SCC-79B	parotid	2.5 ± 0.1	yes	deletion, frameshift	wt	2	2	1	1	1

**Fig. 3:**
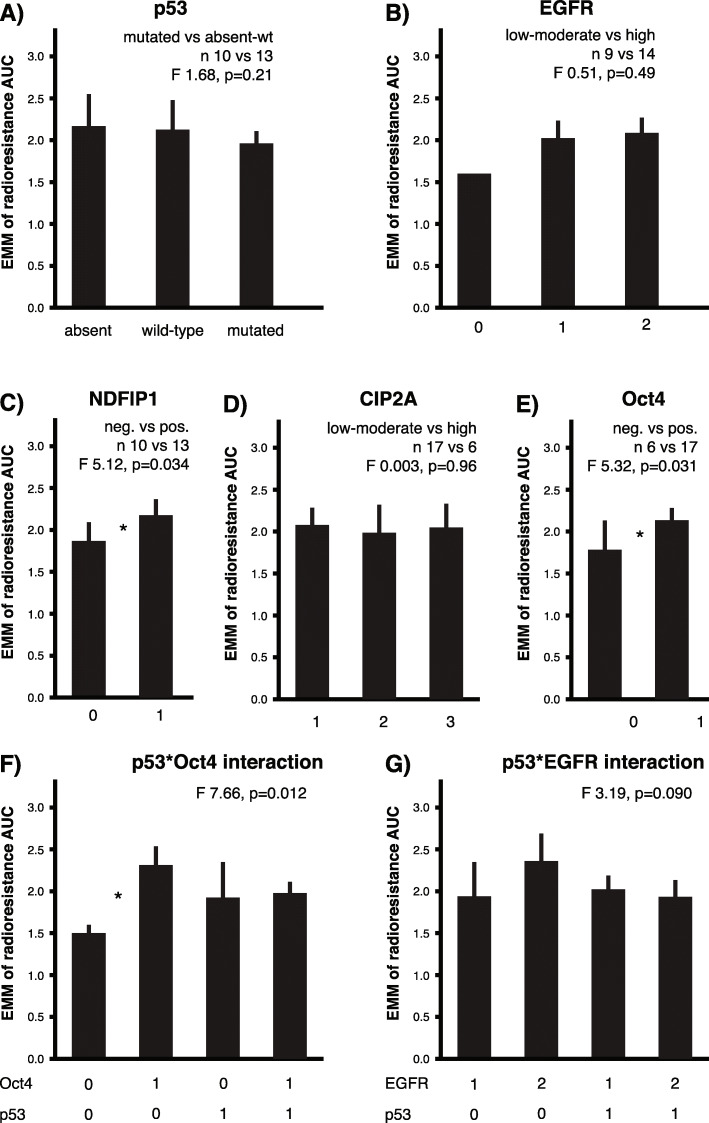
Association of biomarker stains and radioresistance was investigated across 23 cell lines. Bars represent estimated marginal means and error bars indicate 95% confidence intervals determined using bootstrapping. Sample sizes, F values and *p* values are indicated. **A** p53, **B** EGFR, and **D** CIP2A demonstrate no association with radioresistance, whereas **C** NDFIP1, and **E** Oct4 have a significant (*) association with the intrinsic radioresistance of the cell lines. **F** A highly significant interaction effect between p53 and Oct4 was revealed, implying a predictive role of Oct4 in the absence of p53 mutation/amplification type staining. **G** The interaction trend between p53 and EGFR did not reach significance

The associations between staining results and intrinsic radioresistance of the cell lines were analyzed. Expression of p53, EGFR, and CIP2A were not associated with radioresistance (Fig. 3A-B and D). Interestingly, nuclear NDFIP1 expression was associated with a significant increase in radioresistance (Fig. 3C). In accordance with our previous findings, also Oct4 expression was associated with a significant increase in radioresistance (Fig. 3E). Interaction effects of the biomarkers were analyzed to further explore the possible links between different potential radioresistance mechanisms. There was a significant interaction effect between Oct4 and p53 (Fig. 3F). A trend for a comparable interaction effect between p53 and high EGFR expression was noted (Fig. 3G).

### Analysis of CIP2A-shRNA-silenced cell lines

**Fig. 4:**
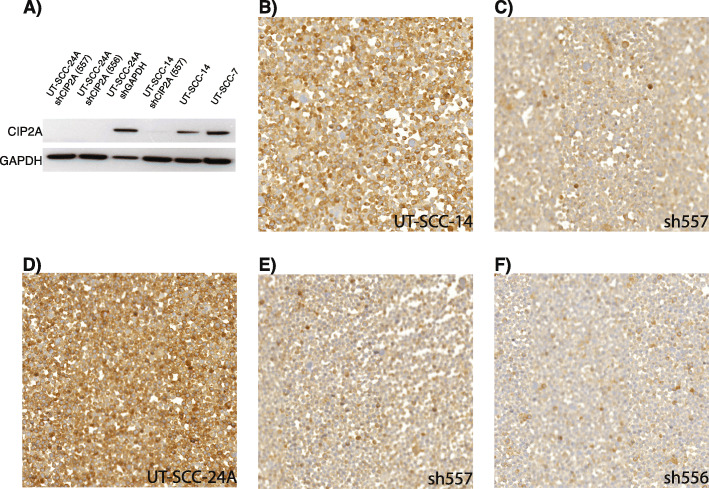
Analysis of CIP2A-shRNA-silenced cell lines. **A** Succesful silencing was confirmed using Western blot. **B-C** Loss of CIP2A immunohistochemical staining in shRNA-silenced cell line in comparison with the non-silenced cell line UT-SCC-14. **D-F** Loss of CIP2A immunohistochemical staining in both shRNA-silenced cell line versions of UT-SCC-24A

To test the functionality of CMA in the validation of antibody specificity, and to study protein interactions in genetically modified cancer cell lines, three CIP2A shRNA-silenced cell lines were included in the CMA. CIP2A shRNA-silencing was confirmed by Western blot (Fig. 4A) and is demonstrated by the minimal immunoreactivity of the silenced cell lines compared to the parental non-silenced lines (Fig. 4B-F). Parallel immunohistochemistry of the potential radioresistance biomarkers did not reveal significant correlations between CIP2A silencing and p53, EGFR, NDFIP1, or Oct4 expression (Table 3).

**Table 3:** Immunohistochemistry of UT-SCC-14 and UT-SCC-24A and corrresponding CIP2A-shRNA-silenced cell lines

Cell line	Immunohistochemistry							
	CIP2A	p53	LIMA1	EGFR	NDFIP1	Oct4	SET	PME-1
Non-treated UT-SCC-14	2	mutated	1	3	0	1	2	2
CIP2A-shRNA-silenced UT-SCC-14 (plasmid 557)	1	mutated	1	3	0	1	1	1
Non-treated UT-SCC-24A	3	absent	2	3	1	1	1	2
CIP2A-shRNA-silenced UT-SCC-24A (plasmid 556)	1	absent	2	3	0	1	1	1
CIP2A-shRNA-silenced UT-SCC-24A (plasmid 557)	1	absent	2	3	1	1	1	1

Since CIP2A failed to predict radioresistance in the CMA and since CIP2A silencing did not affect other putative biomarkers expression, we were interested, whether CIP2A would have an association with other PP2A inhibitors and performed immunohistochemistry of two well-established endogenous PP2A inhibitors, PME-1 and SET, which are not pronouncedly identified as radiotherapy biomarkers (Supplemental Figure 1). Surprisingly, in all three silenced cell lines the expression of both PME-1 and SET was reduced as well, suggesting a CIP2A-mediated circuitry leading to a more universal loss of PP2A inhibitor expression (Table 3). However, CIP2A, PME-1 and SET expression levels were not correlated across other UT-SCC cell lines of the CMA. Neither PME-1 nor SET was associated with intrinsic radioresistance of the cell lines.

## Discussion

HNSCC consists of a genetically and behaviorally heterogeneous group of malignancies. The observed genomic instability is due to mutagenic insults on the mucosal lining of the upper aerodigestive tract such as tobacco and alcohol [25]. Accordingly, HNSCC-derived cell lines exhibit highly variable genomic changes [26–29]. The field cancerization phenomenon makes radiotherapy an especially inviting treatment option, and it is included in the treatment plan of nearly one half of head and neck cancer patients [30]. Since no biomarkers can currently be used to identify patients benefitting of different treatment strategies, or to explain the clinical diversity in radiotherapy outcomes, the question of radioresistance identification is of tremendous importance for treatment of HNSCC.

Prior studies presenting similar methods of cell line array construction have mostly included relatively small amounts of cell lines, and have been constructed with other than head and neck cancers [6, 31, 32]. To our knowledge, this is the first CMA constructed with primarily HNSCC cell lines to this large extent. CMA is a practical, cost-efficient and time-saving way to determine the expression of a protein of interest using a specific antibody in a large number of cell lines simultaneously. In this study, we demonstrate that formalin-fixed, paraffin-embedded cell lines may be handled with similar staining protocols used for clinical samples. Cells retained their morphology and overall cellular architecture, leading to good quality staining result, allowing for a reliable evaluation of IHC staining intensity. Loss of CMA spots is low and comparable to TMA methods.

In addition to efficiency, CMA provides other notable benefits as well. Use of exclusively cellular material allows strictly focusing the evaluation of IHC staining positivity on cancer cells and removing the majority of unspecific sources of unwanted IHC signaling positivity. This also provides a fixed, paraffin-embedded biobank of cell lines. It is possible to manipulate and modify the cell lines before constructing CMA, allowing e.g. molecular profiling of cell lines by inhibiting or enhancing the expression or effect of certain genes. This may also be applied to drug development and discovery studies, since cell lines may be exposed to drug of interest during culture. Moreover, in a CMA, all types of analysis available for FFPE patient samples can be used. As the CMA paraffin block is handled similarly to FFPE samples, cell line results can directly be compared with patient staining results. Our UT-SCC CMA included three CIP2A-shRNA-silenced cell lines, which demonstrate a highly successful silencing after lengthy cell line culture. CMA offers a powerful tool for the characterization of cellular effects of targeted silencing.

A major weakness of cell line experiments in general and thus also of this study, is that cell line models are unable to take into account patient-related factors such as immunological responses, that may decisively affect the success of radiotherapy in HNSCC patients. However, the intrinsic radioresistance of the cancer cells is an important phenomenon in clinical radioresistance [1, 9, 22]. In the present study, expression of the radioresistance-associated Oct4 and NDFIP1 in patient-derived UT-SCC cell lines could not be explained by correlation with clinical factors, supporting the notion, that radiotherapy response cannot be sufficiently predicted by analysis of the patient clinical parameters alone. Interestingly, other putative radioresistance biomarkers – p53, EGFR, and CIP2A – failed to be associated with intrinsic radioresistance of the cell lines in this relatively large panel of HNSCC cell lines.

Oct4 is a transcription factor essential for pluripotency in embryonic stem cells and testicular maturation. Oct4-related stemness has been found to have a role in radiotherapy resistance of various cancers, including HNSCC [13, 14, 33–35]. Especially interesting, regarding HNSCC, is that Oct4 expression is universally acknowledged to be a favourable biomarker for cisplatin sensitivity in testicular cancer, but seems to predict poor cisplatin response in other cancers [36–38]. Since radioresistance is one of the most important issues in the treatment of HNSCC, further studies should investigate the potential role of Oct4-related stemness in the clinical radiosensitivity and chemoradiosensitivity of HNSCC. Furthermore, since Oct4 is expressed by a minority of cells as predicted by the cancer stem cell hypothesis, especially Oct4 overexpression experiments would provide essential information on the relationship of therapy resistance and stemness-like characteristics.

## Conclusions

This study, in addition to confirming the feasibility of CMA methodology to screen for new predictive factors in head and neck cancer, revealed potential immunohistochemical biomarkers associated with radioresistance in head and neck cancer cell lines. Especially interesting for its potential clinical implications is the stem cell marker Oct4 which has been demonstrated to have a significant predictive role in previous studies [13, 14]. In conclusion, our parallel immunohistochemical analysis of 29 HNSCC cell lines suggests, that radioresistance of HNSCC is regulated by stemness-related mechanisms. The relatively straightforward Oct4 immunohistochemical staining might offer a novel and intriguing way to identify cancer radioresistance in HNSCC.

## Supplementary Information

**Additional file 1: Figure S1.** Representative examples of low and moderate PME-1 and moderate and strong SET stainings.

## Data Availability

All data generated or analyzed during this study are included in this published article.
