# Supplementary figures and images for: Cancer cell line microarray as a novel screening method for identification of radioresistance biomarkers in head and neck squamous cell carcinoma
Source: BMC Cancer. 2021 Jul 29;21:868. doi: 10.1186/s12885-021-08618-6 (PMC8320194; doi:10.1186/s12885-021-08618-6)

**Supplemental Figure 1.**

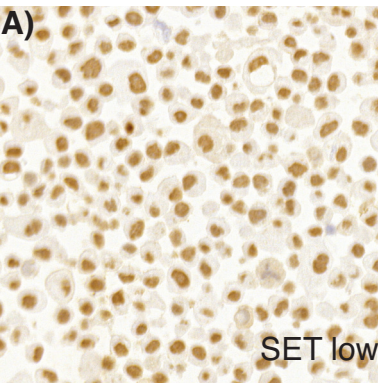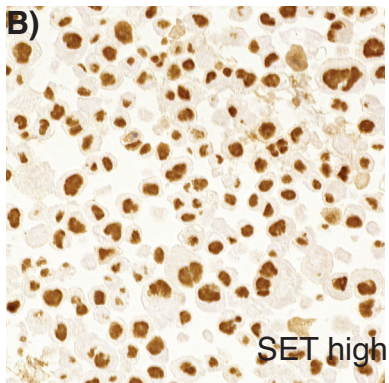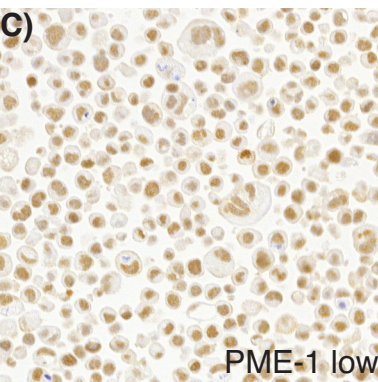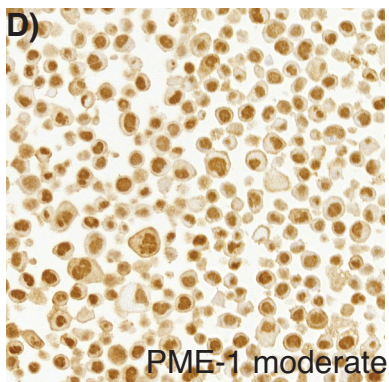

Supplement: Supplementary file 1 — Additional file 1: Figure S1. Representative examples of low and moderate PME-1 and moderate and strong SET stainings. [file 12885_2021_8618_MOESM1_ESM.pdf]
